# Deeper and Deeper on the Role of BK and Kir4.1 Channels in Glioblastoma Invasiveness: A Novel Summative Mechanism?

**DOI:** 10.3389/fnins.2020.595664

**Published:** 2020-11-30

**Authors:** Federico Brandalise, Daniela Ratto, Roberta Leone, Federico Olivero, Elisa Roda, Carlo Alessandro Locatelli, Maria Grazia Bottone, Paola Rossi

**Affiliations:** ^1^Department of Fundamental Neurosciences (NEUFO), University of Geneva, Geneva, Switzerland; ^2^Department of Biology and Biotechnology “L. Spallanzani,” University of Pavia, Pavia, Italy; ^3^Pavia Poison Centre, National Toxicology Information Centre, Laboratory of Clinical & Experimental Toxicology, Toxicology Unit, Istituti Clinici Scientifici Maugeri IRCCS, Pavia, Italy

**Keywords:** glioblastoma, Kir4.1, BK channel, cancer, channelopathy

## Abstract

In the last decades, increasing evidence has revealed that a large number of channel protein and ion pumps exhibit impaired expression in cancers. This dysregulation is responsible for high proliferative rates as well as migration and invasiveness, reflected in the recently coined term oncochannelopathies. In glioblastoma (GBM), the most invasive and aggressive primary brain tumor, GBM cells modify their ionic equilibrium in order to change their volume as a necessary step prior to migration. This mechanism involves increased expression of BK channels and downregulation of the normally widespread Kir4.1 channels, as noted in GBM biopsies from patients. Despite a large body of work implicating BK channels in migration in response to an artificial intracellular calcium rise, little is known about how this channel acts in GBM cells at resting membrane potential (RMP), as compared to other channels that are constitutively open, such as Kir4.1. In this review we propose that a residual fraction of functionally active Kir4.1 channels mediates a small, but continuous, efflux of potassium at the more depolarized RMP of GBM cells. In addition, coinciding with transient membrane deformation and the intracellular rise in calcium concentration, brief activity of BK channels can induce massive and rapid cytosolic water loss that reduces cell volume (cell shrinkage), a necessary step for migration within the brain parenchyma.

## Introduction

Glioblastoma (GBM, WHO grade IV astrocytoma) is the most common and malignant brain tumor (Brat et al., 2007; Furnari et al., 2007; Barbieri et al., 2018). In comparison to the majority of solid tumors, it is characterized by strong invasive and pro-angiogenic behavior associated with a poor prognosis with a median survival rate of about 15 months (Stupp et al., 2009). Because of the diffuse and aggressive invasiveness of GBM, it is generally not possible to achieve complete surgical resection, resulting in rapid relapse (Holland, 2001; Maher et al., 2001). Furthermore, GBMs contain a small subpopulation of so-called cancer stem cells (CSCs), which are extremely resistant to radio and chemotherapy (Vescovi et al., 2006; Lathia et al., 2011). Death evasion minimizes the effect of all therapeutic strategies currently available, but enhanced invasiveness is the major feature that prevents successful treatment (Louis et al., 2007).

## Glioblastoma as a Channelopathy: the Role of Chloride and Potassium Ionic Equilibrium in GBM Cell Invasiveness

Glioblastoma cells migrate through narrow spaces in the brain parenchyma (De Vleeschouwer and Bergers, 2017) that are usually smaller than the soma of the cell (around 8 μm in diameter, Liu et al., 2018). To make that possible, GBM cells undergo a reduction in their volume of about 30% facilitating migration and invasion (Demuth and Berens, 2004; Armento et al., 2017). Such shrinkage is achieved by modifying the osmotic equilibrium in the cell allowing a net release of cytoplasmic water. Increasing evidence suggests that specific ion channels and transporters are involved in modulating the cell volume. The two main ionic gradients reported to be altered in glioma cells are for chloride (Cl^–^) and potassium (K^+^) ions (Turner et al., 2014).

In contrast to other neurons in the brain, glioma cells have a higher cytosolic Cl^–^ concentration (Habela et al., 2009). The chloride gradient is maintained and modulated by persistent activity of the Na^+^/K^+^/Cl^–^ cotransporter 1 (NKCC1), hence its expression has been linked with GBM invasiveness and severity grade (Garzon-Muvdi et al., 2012). The overexpression of the NKCC1 cotransporter leads to abnormal accumulation of Cl^–^ in the glioma cell cytosol (Haas and Sontheimer, 2010; Ben-Ari, 2017) so that upon opening of Cl^–^ channels (Barbieri et al., 2018), the altered electrochemical gradient results in an outward flow of this ion with concomitant osmotic loss of water from the intracellular milieu (through the aquaporin channels). The net result is a loss of cellular volume (Luo et al., 2020).

Along with Cl^–^ gradient, the altered K^+^ flux, is also essential for invasion. In fact, due to the cytosolic calcium fluctuations during GBM cells migration, members of the family of Ca^2+^-activated K^+^ channels such as KCa3.1 (intermediate conductance K^+^ channel) and the BK channel (large conductance K^+^ channel), are overexpressed in 32% of glioma patients, and there is a linear correlation between the expression of these channels and the progression of the pathology. Due to their calcium sensitivity, it has been shown that such channels respond positively to bradykinin activation that increases intracellular Ca^2+^, with a resulting efflux of K^+^ and water (Reetz and Reiser, 1996; Catacuzzeno and Franciolini, 2018). As a consequence, the glioma cells reduce their total volume, which enables them to migrate through narrow spaces within the brain.

While the importance of the intermediate conductance K^+^ channel in GBM progression has been recently summarized in several works (Catacuzzeno et al., 2012; D’Alessandro et al., 2018; Liu et al., 2019), the aim of this review is to underline the contributions of BK channels and Kir4.1 channels on GBM invasiveness, focusing on their biophysical properties and their osmo-electric effect at the RMP in GBM cells.

## The BK Channel: From Structure to Physiology in Glioblastoma Channelopathy

The BK channel is a tetrameric, large conductance K^+^ channel, widely expressed in both neurons and glia across development and adulthood (for an extensive review see Lee and Cui, 2010). The BK channel is characterized by an outwardly rectifying current that shows both voltage and calcium-concentration sensitivity (Nardi and Olesen, 2008; Cui et al., 2009).

Overexpression of this channel has been reported in biopsies of glioblastoma patients (Liu et al., 2002; Catacuzzeno et al., 2015) and intriguingly the channel structure also seems to be altered, since Ransom et al. (2002) reported that GBM cells expressed a splicing variant of the channel on the *hSlo* (the gene linked to the encoding part of the alpha subunit) with a consequent increase in the sensitivity to intracellular calcium concentration.

Intracellular calcium dynamics are involved in the regulation of a wide number of processes in the brain that span from synaptic plasticity (Brandalise and Gerber, 2014; Brandalise et al., 2016a; Keck et al., 2017) to remodeling of cytoskeleton (Lebart and Benyamin, 2006; Correll et al., 2008). In GBM it has been demonstrated that calcium fluctuations from the intracellular stores (reticulum) along with different states of the RMP (depolarized versus hyperpolarized) are linked to GBM cell migration as well as to the proliferative state of the cell (Rondé et al., 2000; Ishiuchi et al., 2002; Catacuzzeno et al., 2011). Cyclic variation of both voltage and calcium concentration in GBM cells has led to the hypothesis that BK channels, in light of their overexpression, can be one of the key targets in triggering glioblastoma migration and the invasion process (Catacuzzeno et al., 2015).

## Trying to Explain the BK Role at the GBM RMP

The implication of BK channels in GBM cell migration and invasion has been reported by various groups (Klumpp et al., 2018; Rosa et al., 2018). Blockade of BK channels with iberiotoxin (IbTx) or tetraethylammonium (TEA) in two- dimensional migration assays inhibits GBM cell motility (Soroceanu et al., 1999; Basrai et al., 2002; Weaver et al., 2006). Furthermore, the increase of intracellular calcium induced by extracellular menthol application significantly increases BK current and the migration of GBM cells and this effect was reversed by BK channel blockers (Wondergem and Bartley, 2009; Ratto et al., 2019).

However, despite the known functional upregulation of BK channels in GBM, their dependence on membrane potential deserves further examination. At resting cytoplasmic free calcium concentration (10–100 nM), BK channels open only at membrane potentials above +10 mV (Lee and Cui, 2010), significantly more depolarized than the RMP of around −40 mV measured in GBM cells (Catacuzzeno et al., 2015). In other words, BK channels at RPM are in the closed state. Additionally, in the two-dimensional migration assay, there is a general consensus that blockade of BK channels does not significantly reduce migration of GBM cells. Nevertheless, the reduction of GBM cell invasion due to BK pharmacological blockade is effective only when the intracellular calcium concentration is raised (for example, by menthol or acetylcholine bath application) (Bordey et al., 2000; Kraft et al., 2003; Wondergem and Bartley, 2009). Therefore, although activating BK channels undoubtedly boosts GBM cell migration due to K^+^ efflux, the blockade of the same conductance under resting conditions does not prevent GBM invasiveness.

## Kir4.1 in Globlastoma: Primum Movens of RMP Depolarization in GBM Cells?

**FIGURE 1:**
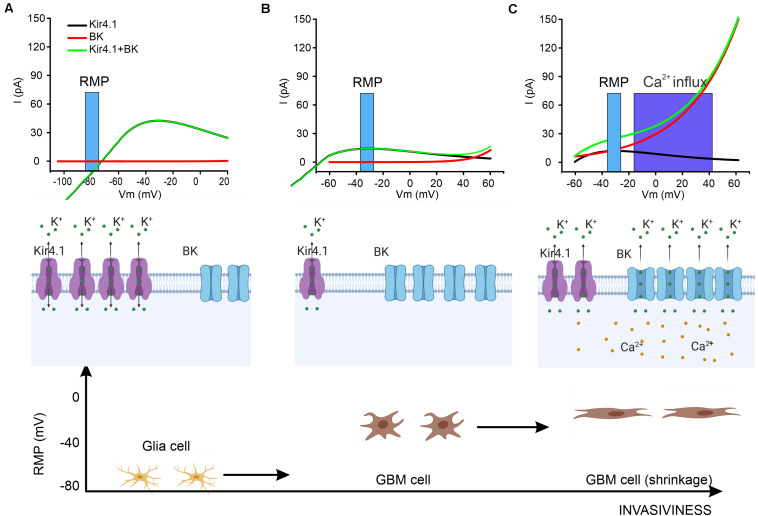
A proposed action mechanism for BK and Kir4.1 channels. **(A)** In glial cells the RMP is set at hyperpolarized potentials so that Kir4.1 exerts a homeostatic equilibrium whereas BK channels are in the closed state. The **top panel** shows the kinetics of BK and Kir4.1 channel in the range of the RMP (blue bar). **Middle panel:** schematic of the expression of the two channels and the direction of the ionic potassium flow (K^+^). **Bottom panel:** representation of glia cells in relationship with their RMP. **(B)** When glia transforms into GBM cells, the RMP shifts to a more depolarized potential **(top and bottom)** due to a downregulation of Kir4.1 channels while BK channels are overexpressed **(middle)**. **(C)** The increasing pressure on the GBM cells membrane during migration in the brain parenchyma exerts a double effect by directly activating BK channels and by raising the concentration of cytosolic calcium. This results in the opening of BK channels **(top and middle)** with consequent cell shrinkage **(bottom)** allowing the migration of the GBM cell.

Kir4.1, one of the inwardly rectifying potassium channels (coded by the KCNJ10 gene) is largely expressed in the glia cells of the brain (Nichols and Lopatin, 1997). In mature astrocytes, the high potassium permeability is mediated to a large extent by the Kir4.1 channel with two functional consequences: first, the negative RMP that is closer to the potassium equilibrium potential, and, second, the buffering of extracellular potassium after neuronal activity (Butt and Kalsi, 2006; Chever et al., 2010). Because a fraction of the channels is constitutively open, it has been proven that Kir4.1 plays a role in the homeostatic regulation of the RMP : for example, this is a crucial mechanism in cerebellar granule cell development during migration (Rossi et al., 1998; Brandalise et al., 2016b) as well as for glia maturation that settles the RMP at around −80 mV (Olsen and Sontheimer, 2008). Their peculiar current/voltage relation (Figure 1A) is due to weak, voltage-dependent rectification and to voltage-dependent pore block, from the internal side of the channel, by magnesium and other organic cations such as polyamine (Ruppersberg, 2000). At potentials more positive to the K^+^ equilibrium, this block limits the amount of K^+^ that flows through the channel. Kir4.1 is also involved in the regulation of other pathways in astrocytes such as the BDNF expression (Ohno et al., 2018).

In GBM, a downregulation of Kir4.1 during the early stage of the tumor progression has been reported (Olsen and Sontheimer, 2004; Ratto et al., 2019), which is correlated to the dramatic shift in RMP of GBM cells to more depolarized values around −40/−30 mV (Olsen and Sontheimer, 2004). Due to their internalization during GBM progression, very little attention has been paid to the residual activity of the Kir4.1 channels at RMP. However, recent investigations have provided evidence that justifies a reconsideration of the impact of this channel on the later progression of GBM:

• Despite the internalization and consequent functional downregulation of Kir4.1, there is still a significant fraction of this channel expressed in the membrane which, at the RMP of GBM cells, mediates a constitutive outward K^+^ current (Figure 1B) that might play a role in the redistribution of cell volume and the consequent change in cell morphology.
• The fact that the channels are internalized but not degraded suggests that they still have a functional role as a readily available pool that is potentially re-inserted into the membrane in a relatively short time. Indeed, despite the fact that the mechanism is not yet understood, in Ratto et al., 2019, a calcium-dependent upregulation of Kir4.1-mediated current has been described within 9 min of menthol bath-application.
• The simultaneous block of Kir4.1 along with the BK channel seems to be an effective strategy for blocking GBM cell invasiveness in two-dimensional migration assays without additional perturbation (raising) of the cytosolic calcium concentration (Ratto et al., 2019). This suggests that the two classes of potassium channels are mutually involved in the shrinkage of the cell via regulation of the K^+^ gradient.

## Kir4.1 and BK Channels: A Summative Link?

In the previous sections, we have summarized the literature on the role of BK and Kir4.1 channels in GBM invasiveness. We have linked the physiology of these two channels relating to the microenvironment of GBM cells (with particular interest in the K^+^ electrical and osmotic gradient) raising some discrepancies between the channel biophysical properties and the electrochemical equilibrium range of GBM cells.

Now we tentatively propose how these two channels might work in concert during GBM cell invasion:

(1) Glia cells under physiological conditions: glia cells express a fairly high level of Kir4.1 that is known to be involved in ionic homeostasis by buffering ambient K^+^ during neuronal activity. Moreover, glia cells have a strongly hyperpolarized RMP maintained mainly by Kir4.1 (the reversing potential of Kir4.1 in normal conditions is around −75/−80 mV). BK channels, on the other hand, are also expressed but are not activate at in the RMP (Figure 1A).
(2) Morphological changes in GBM cells: the severe alterations in Cl^–^ and K^+^ equilibrium in the cells prior to migration leads to a more depolarized RMP (−40 to −30 mV). Consequently, the reversal potential of Kir4.1 is no longer aligned with the RMP, and this sets the channel for a constitutively net outflow of K^+^ ions (see Figure 1B). It is worth noting that in basal conditions, GBM cells seem to change their shape due to the efflux of water, but the total surface area of the cell is actually not reduced, probably due to the formation of lamellipodia. In this phase, BK channels are overexpressed in their spliced isoforms, which increases their sensitivity to the calcium concentration. Therefore, despite the fact that the RMP is still below the BK threshold, transient cytosolic calcium rise (Catacuzzeno et al., 2011; Li et al., 2020) could depolarize the cell and shift the activation curve to a more hyperpolarized potential allowing a transient opening of BK channels (Figure 1C). Under these conditions, BK channels can contribute efficiently and summate with Kir4.1 channels in controlling total efflux of potassium and consequent shrinkage. This scenario agrees with recent experimental evidence whereby blocking Kir4.1 and BK channels reduces GBM cell migration.
(3) Glioblastoma invasiveness in the brain parenchyma: When a GBM cell invades the extracellular matrix in the brain, the process requires a reduction of cytosolic volume. The increase in pressure that the extracellular matrix exerts on the GBM cell induces mechanical stress on the membrane, which has been suggested to activate BK channels (Zhao et al., 2010; Wawrzkiewicz-Jałowiecka et al., 2018). Moreover, this mechanical constraint might lead to a possible biochemical cascade with a consequent increase of intracellular calcium concentration that shifts the voltage activation curve of the channel to a more hyperpolarized potential (Charles et al., 1991). Overall, increased pressure on the GBM cell membrane results in greater functional BK channel activation that significantly increases the efflux of cytosolic water with consequent additional reduction in volume and capability to migrate efficiently (Figure 1C). Following the mechanical compression, the relief of pressure reduces the fraction of active BK channels and the balance of activation between the two channels returns to the state described in point 2 (Figure 1B).

## Conclusion

This review has not only the purpose of summarizing the most recent evidence on the role of BK and Kir4.1 channels in GBM cell-migration, but underlines the need for examining the roles of these channels in the context of the different functional states of GBM cells. The emerging perspective that considers GBM as a channelopathy is a promising field (Litan and Langhans, 2015; Prevarskaya et al., 2018). However, many questions must be yet addressed for a more comprehensive understanding. The intracellular calcium concentration and its spontaneous oscillations seem to be the main actor behind the scenes as it modulates a large fraction of the ion channels implicated in GBM invasion. Furthermore, recent work has proposed that BK channels are modulated by mechanical stress on the membrane indicating that the dynamics of GBM cell membrane changes must be investigated. Finally, K^+^ equilibrium is a result of the interactions between a large variety of channels and pumps; in this review we focused on BK and Kir4.1 on account of their high conductance and their roles at RMP, respectively. However, for deeper insight into GBM cell invasiveness, future investigations should take into account other channels as well, such as intermediate conductance calcium-activated potassium channels and leak channels. In conclusion, a large number of potassium channels play a key role in GBM progression. However, despite that potassium channels offer a surface-accessible therapeutical target, the fact that they are largely expressed in other cell types such as neurons, glia and cardiomyocytes imposes a strong monitoring of the acceptable toxicity threshold induced by potentials specific blockers as a side effect.

## Author Contributions

FB and PR: study concepts and design. FB, PR, ER, and DR: manuscript preparation. FO and CL: manuscript editing. RL and MG: manuscript review. All authors contributed to the article and approved the submitted version.

## Conflict of Interest

The authors declare that the research was conducted in the absence of any commercial or financial relationships that could be construed as a potential conflict of interest.
